# Immunoadsorption therapy for chronic inflammatory demyelinating polyradiculoneuropathy in a patient on maintenance hemodialysis: A case report

**DOI:** 10.1097/MD.0000000000050038

**Published:** 2026-07-31

**Authors:** Changqin Zhang, Songtao Niu, Yilun Zhou

**Affiliations:** a Department of Nephrology, Beijing Tiantan Hospital, Capital Medical University, Beijing, China; b Department of Neurology, Beijing Tiantan Hospital, Capital Medical University, Beijing, China.

**Keywords:** chronic inflammatory demyelinating polyradiculoneuropathy, efficacy, immunoadsorption, maintenance hemodialysis

## Abstract

**Rationale:**

Chronic inflammatory demyelinating polyradiculoneuropathy (CIDP) is a rare immune-mediated disorder that primarily targets the myelin sheaths of peripheral nerves, leading to limb weakness and sensory disturbances. Established treatments for CIDP include glucocorticoids, intravenous immunoglobulin, and plasma exchange. Immunoadsorption (IA) has emerged as a promising alternative to plasma exchange and has shown favorable efficacy in patients who respond inadequately to first-line therapies.

**Patient concerns:**

A 37-year-old man presented with progressive numbness and weakness of the limbs. He was a hemodialysis patient with type 2 diabetes mellitus and recurrent buttock furuncles. Subsequent electrophysiological testing indicated demyelinating lesions in 2 peripheral nerves.

**Diagnoses:**

The diagnosis of CIDP was based on clinical symptoms and electrophysiological examinations.

**Interventions:**

IA was selected as the initial monotherapy and long-term maintenance therapy.

**Outcomes:**

This case demonstrated that IA was well tolerated and associated with sustained clinical improvements.

**Lessons:**

The use of IA for both initial and maintenance therapy may represent an optimal individualized treatment for patients with CIDP undergoing hemodialysis.

## 1. Introduction

Chronic inflammatory demyelinating polyradiculoneuropathy (CIDP) is an immune-mediated neurological disorder. Most patients with CIDP are male, and both incidence and prevalence increase with age.^[1]^ CIDP is diagnosed based on a combination of clinical manifestations and electrophysiological findings. It is typically characterized by slow progression, symmetrical involvement, proximal and distal muscle weakness, sensory dysfunction, and symptom duration exceeding 8 weeks. The disease course may either be progressive or recurrent.^[2]^

Electrophysiological evidence of demyelination in peripheral motor nerves is required for the diagnosis of CIDP. This includes prolonged distal latency, reduced motor conduction velocity, prolonged F-wave latency, and partial motor conduction blocks. These abnormalities must be present in at least 2 nerves to confirm the diagnosis.^[3]^ Most patients experience marked functional improvement with immunotherapy; however, these responses are often transient, and several patients require maintenance treatment for years or even decades. A subset of patients with CIDP does not respond well to treatment and may progress to severe neurological dysfunction.^[1,4]^

Glucocorticoids, intravenous immunoglobulin (IVIG), and plasma exchange (PE) are preferred treatment modalities.^[2]^ Studies have suggested that immunoadsorption (IA) may be effective for patients with CIDP who exhibit an inadequate response to first-line therapies. However, data remain limited regarding the use of IA as both an initial monotherapy and long-term maintenance therapy for patients with CIDP, particularly those undergoing maintenance hemodialysis (MHD). Here, we report a case of a patient with CIDP on MHD who demonstrated an excellent response to IA-based induction and maintenance therapy, with no observed complications.

## 2. Case report

A 37-year-old male with a 5-year history of type 2 diabetes mellitus and recurrent buttock furuncles had an elevated serum creatinine level (130–140 μmol/L), first noted in 2020. Renal biopsy confirmed a diagnosis of IgA nephropathy. In February 2023, he initiated hemodialysis (HD) via a left forearm arteriovenous fistula after his serum creatinine rose to over 1300 μmol/L. He was on thrice-weekly HD, with good dialysis tolerance and adequate dialysis efficiency (Kt/V: 1.3–1.4). At that time, he also began experiencing numbness in both lower extremities, more pronounced on the left side.

Six months later, the patient developed progressive lower limb weakness, became unable to stand independently, and required a wheelchair for mobility assistance. Numbness and weakness later developed in both upper extremities, affecting his ability to perform fine motor tasks such as writing or holding objects. Muscle strength in both lower extremities continued to decline, and eventually, he could not lift his legs from the bed. Throughout the disease course, he did not experience headache, vomiting, visual disturbances, incontinence, respiratory symptoms, or altered consciousness.

Upon admission in December 2023, physical examination revealed the following: bilateral proximal upper limb strength, grade 5; distal left upper limb, grade 3; distal right upper limb, grade 4; bilateral proximal lower extremities, grade 3; and bilateral distal lower extremities, grade 2. Grip strength was significantly reduced bilaterally, and reflexes (biceps, triceps, knee, and Achilles tendon) were absent. Sensory deficits were present in the distal extremities. The Inflammatory Neuropathy Cause and Treatment (INCAT) score was 7, the Inflammatory Rasch-built Overall Disability Scale (I-RODS) score was 21, and the Medical Research Council Sum Score (MRCSS) was 54.

Laboratory tests showed a hemoglobin level of 107 g/L and an erythrocyte sedimentation rate of 37 mm/h. The serum creatinine level was 745 μmol/L, blood urea nitrogen was 19.2 mmol/L, and glycated hemoglobin was 7.3%. Other laboratory parameters, including blood electrolytes, liver, and thyroid function, were all within normal limits. C-reactive protein, serum monoclonal protein, and autoantibodies related to autoimmune diseases also showed no abnormalities. Chest computed tomography, cranial magnetic resonance imaging, and thoracolumbar spinal magnetic resonance imaging demonstrated no pathological changes. Glycemic monitoring indicated that the patient’s fasting blood glucose fluctuated between 6 and 7 mmol/L, and postprandial blood glucose ranged between 8 and 10.5 mmol/L.

Electrophysiological testing indicated peripheral neuropathy in both upper and lower extremities, with conduction block in the right median (elbow–wrist segment) and right ulnar (below elbow–wrist segment) nerves. Sympathetic nerve dysfunction was present, but parasympathetic nerve dysfunction could not be excluded. The final diagnosis was CIDP with overlapping uremic and diabetic peripheral neuropathy.

Owing to financial concerns, the patient refused treatment with IVIG. Given the patient’s end-stage renal disease complicated by type 2 diabetes mellitus and recurrent buttock furuncles, IA was chosen for induction therapy due to its favorable safety profile. The patient had long-term arteriovenous fistula access, which also made IA feasible for maintenance therapy. We conducted IA followed by HD on the same day. The process commenced with approximately 2 hours of IA and subsequently proceeded with 4 hours of HD. Low-molecular-weight heparin was administered intravenously 30 minutes prior to each IA session to prevent coagulation. Blood was drawn using the Plasauto-Σ blood purification system (Plasauto-Σ, Asahi Kasei Medical Co., Ltd., Tokyo, Japan), and the OP-08W membrane plasma separator (OP-08W, Asahi Kasei Medical Co., Ltd., Tokyo, Japan) was used to fractionate whole blood into plasma and cellular components, along with the immunoadsorber IMMUSORBA TR-350 (TR-350, Asahi Kasei Medical Co., Ltd., Tokyo, Japan). This type of adsorption column uses tryptophan, a typical hydrophobic amino acid, as the ligand. It binds pathogenic substances, including various antibodies, via electrostatic and hydrophobic interactions. It has a substantial capacity to adsorb immunoglobulin G subclasses 1 and 3. A total of 2.5 L of plasma was processed per session. One IA course consisted of 4 sessions administered every other day.

Following the first IA course in December 2023, the patient was discharged with oral vitamin B1 and mecobalamin. In April 2024, he was readmitted for a second IA course, again consisting of 4 alternate-day sessions. Compared with the previous visit, muscle strength had improved: right lower limb proximal strength increased from grade 3 to grade 4, and distal strength improved from grade 2 to grade 3. He then began receiving IA twice monthly.

By August 2024, further improvements were noted: distal left upper limb strength increased from grade 3 to grade 4; proximal left lower limb strength increased from grade 3 to grade 4; and distal left lower limb strength increased from grade 2 to grade 3. The INCAT, I-RODS, and MRCSS scores were 6, 26, and 64, respectively. From October 2024 onward, IA was administered twice every 6 weeks. By March 2025, strength in both distal lower limbs had improved from grade 3 to grade 4. The INCAT, I-RODS, and MRCSS scores were 4, 34, and 70, respectively – representing gains of 3, 13, and 16 points over 16 months. Since May 2025, IA has been administered twice every 2 to 3 months, and the improvement in muscle strength has been maintained. The patient regained the ability to dress and eat without assistance and was capable of short-duration standing with assistive devices. Electrophysiological parameters showed minimal improvement, with only slight changes noted in the right median nerve by July 2024. No significant adverse effects occurred during treatment.

## 3. Discussion

CIDP is an autoimmune polyneuropathy characterized by progressive demyelination of peripheral nerves, which disrupts nerve conduction. Although cycles of demyelination and remyelination are the main pathological features, inflammation may also involve axons, potentially leading to axonal degeneration.^[5]^

The primary goal of CIDP treatment is to achieve disease remission or improve muscle strength and function. Long-term treatment is often necessary to prevent disability. Patient responses vary, and treatment regimens must be tailored to individual needs.^[6]^ CIDP management typically involves induction and maintenance therapy. Glucocorticoids, IVIG, and PE are established first-line treatments for CIDP.^[1,5,7,8]^ Approximately 50% to 80% of patients respond to these treatments.^[4,9–11]^ However, the optimal treatment sequence remains unclear, and several factors – including treatment cost, adverse effects, treatment duration, need for regular hospital visits, and ease of administration – must be considered before initiating therapy.

In recent years, although little progress has been made with glucocorticoids or PE strategies, IA has emerged as a promising alternative. IA is a blood purification technique that enables the selective removal of humoral factors such as immunoglobulin from plasma separated through a high-affinity adsorbent.^[12]^ Compared to PE, IA offers the advantage of selectively removing target substances while preserving beneficial plasma proteins such as coagulation factors.^[13,14]^ Furthermore, it eliminates the need for reinfusion of plasma or albumin, thereby avoiding associated risks such as allergic reactions, intolerance, infections, and coagulopathies.^[15,16]^ Consequently, IA is generally considered a low-risk intervention.

Several studies support the efficacy and safety of IA for the treatment of CIDP. In a randomized trial of 20 patients with CIDP, IA was found to be as effective and as well tolerated as PE, providing valuable evidence for its clinical application.^[15]^ Another randomized study comparing IA and IVIG reported higher clinical response rates in the IA group than in the IVIG group (80% vs 50%), with good overall tolerability.^[17]^ A retrospective study involving 14 patients with CIDP with inadequate responses to first-line therapy found that all 10 hospitalized patients improved significantly after a course of IA (3–5 sessions per course), and all exhibited a significant decrease in INCAT scores. The remaining 4 patients treated as outpatients (1–2 sessions per week) showed improved disability status with ongoing IA maintenance. This study demonstrated that IA treatment is both effective and safe for patients with CIDP who show an inadequate response to first-line or maintenance therapies while avoiding regular supplementation with human plasma products.^[18]^

According to the 2019 American Society for Apheresis guidelines, PE or IA may offer short-term benefit in CIDP; however, relapse is common, and maintenance therapy – whether IA, PE, or other immunomodulatory treatments – is often necessary. Treatment frequency should be adjusted based on individual symptoms and tolerance.^[16]^ A prospective study of 17 patients with CIDP who had inadequate responses to first-line therapy found IA to be a promising option for both short-term (2 weeks) and long-term disease control, with treatment durations extending up to 46 months.^[19]^ However, no prior study has investigated IA as both induction and maintenance therapy in patients with CIDP undergoing MHD.

Patients on MHD face elevated infection risks, and long-term glucocorticoid use may increase the likelihood of adverse outcomes. In the present case, IA was selected based on the patient’s individual profile, including personal financial status, underlying end-stage renal disease, recurrent buttock furuncles, and the presence of long-term vascular access. IA was used for both induction and ongoing maintenance therapy, with the treatment interval gradually extended based on the patient’s therapeutic response. The patient showed progressive improvement in clinical outcomes, with significant gains in INCAT, I-RODS, and MRCSS scores, and experienced no severe adverse effects. Notably, the patient was never administered glucocorticoids, immunosuppressants, or biological agents during treatment.

Although nerve conduction studies are essential for diagnosing CIDP, their value in tracking therapeutic outcomes is debated. Some studies have shown mild improvements in electrophysiological parameters in patients with CIDP following short-term treatment, but these changes may not correspond with clinical outcomes.^[20–22]^ A longitudinal study over 31 years found that electrophysiological improvements could lag behind clinical recovery by up to 2 years, while worsening findings predicted clinical relapse.^[23]^ In our patient, clinical function improved markedly, whereas electrophysiological changes were minimal. This may be related to the lag in electrophysiological response observed in CIDP or the coexistence of metabolic peripheral neuropathies. IA may ameliorate electrophysiological alterations caused by CIDP but has limited efficacy in uremic or diabetic peripheral neuropathy.

The use of IA for both induction and maintenance therapy may represent an optimal individualized treatment for patients with CIDP undergoing MHD. This case demonstrated that IA was well tolerated and associated with sustained clinical improvement, without the need for glucocorticoids or immunosuppressants. Further studies are warranted to systematically assess the efficacy and safety of IA during both treatment phases in patients with CIDP.

## Acknowledgments

We thank the patient for agreeing to the clinical examination and use of data.

## Author contributions

**Conceptualization:** Songtao Niu, Yilun Zhou.

**Writing – original draft:** Changqin Zhang.

**Writing – review & editing:** Songtao Niu, Yilun Zhou.
